# Non-linear association between admission temperature and neonatal mortality in a low-resource setting

**DOI:** 10.1038/s41598-020-77778-5

**Published:** 2020-11-27

**Authors:** Francesco Cavallin, Serena Calgaro, Valentina Brugnolaro, Olivier Manzungu Wingi, Arlindo Rosario Muhelo, Liviana Da Dalt, Damiano Pizzol, Giovanni Putoto, Daniele Trevisanuto

**Affiliations:** 1 Doctors With Africa CUAMM, Padua, Italy; 2 Department of Woman’s and Child’s Health, University of Padova, Via Giustiniani, 3, 35128 Padua, Italy; 3 Central Hospital of Beira, Beira, Mozambique; 4 Unaffiliated, Solagna, Italy

**Keywords:** Risk factors, Paediatric research

## Abstract

Both neonatal hypothermia and hyperthermia represent important risk factors for neonatal mortality, but information on mortality risk across a full range of neonatal temperatures is lacking in low-resource settings. We evaluated the association between neonatal mortality and a full range of admission temperatures in a low-resource setting. This retrospective observational study was conducted at Beira Central Hospital, Mozambique. The relationship between admission temperature and mortality was evaluated using multivariable analyses with temperature modeled as non-linear term. Among 2098 neonates admitted to the Special Care Unit between January–December 2017, admission temperature was available in 1344 neonates (64%) who were included in the analysis. A non-linear association between mortality rate and temperature was identified. Mortality rate decreased from 84% at 32 °C to 64% at 34.6 °C (− 8% per °C), to 41% at 36 °C (− 16% per °C), to 26% to 36.6 °C (− 25% per °C) and to 22% at 38.3 °C (− 2% per °C), then increased to 40% at 41 °C (+ 7% per °C). Mortality rate was estimated to be at minimum at admission temperature of 37.5 °C. In conclusions, the non-linear relationship highlighted different mortality risks across a full range of neonatal temperatures in a low-resource setting. Admission temperature was not recorded in one third of neonates.

## Introduction

Maintaining normothermia at birth is a major challenge for newbosrn survival^1^. Both hypothermia and hyperthermia represent important risk factors for neonatal morbidity and mortality^2^. Neonatal hypothermia is common in both high- and low-resource settings, but is rarely indicated as direct cause of death, while it is associated with a considerable portion of neonatal mortality, mostly as comorbidity of preterm birth, asphyxia and sepsis^3^. Neonatal hyperthermia is associated with brain injury and hemodynamic changes^4^. A recent systematic review including 20,911 participants from 12 studies indicated a 57.2% prevalence of neonatal hypothermia in Eastern Africa^5^. Of note, hypothermia in low-resource settings involves both term and preterm infants^5,6^. In low-middle resource settings, neonatal hypothermia was associated with elevated mortality risk in both hospital and community studies^7–12^.

The World Health Organization (WHO) recommends maintaining neonatal temperature between 36.5 and 37.5 °C, with lower temperatures (< 36.5 °C) defining hypothermia ranges and higher temperatures (> 37.5 °C) defining hyperthermia ranges^13^. Literature offers heterogeneous definitions of neonatal thermal ranges^3^, in search for an optimal classification that mirrors clinical outcomes.

Although offering the advantages of easy interpretation and application in clinical practice, the classification of a continuous variable has the strong disadvantage of underestimating the variation of the outcome^14^. In 2015, the Canadian Neonatal Network attempted to overcome such disadvantages by evaluating a large cohort of very preterm infants where neonatal temperature at admission to neonatal intensive care unit (NICU) was categorized in nine groups with 0.5 °C increments from < 34.5 to ≥ 38.0 °C^15^. The authors suggested a U-shaped relationship between admission temperature and adverse neonatal outcomes, with lowest rate of adverse outcomes between 36.5 and 37.2 °C^15^. The U-shape relationship highlights that both low and high temperatures impair neonatal outcomes, in agreement with international recommendations^2,15,16^.

Maintaining normothermia at birth is an even more crucial challenge in low-resource settings, where appropriate thermal care of the newborn is often neglected due to poor provider training, limited awareness of the problem and limited availability of equipment^3,17^. Furthermore, thermal stability (including warm delivery room, immediate drying, skin-to-skin contact, early breastfeeding, delayed bathing, adequate clothing, warm transport, keeping baby close to the mother) is not a central objective in management protocols in low-resource settings^6,13^. To our knowledge, information on mortality risk across a full range of admission temperatures is lacking in these settings. This study aimed to evaluate the relationship between mortality and a full range of admission temperatures in a low-resource setting.

## Methods

### Study design and setting

This is a retrospective study on the relationship between admission temperature and in-hospital neonatal mortality at the Special Care Unit (SCU) of the Beira Central Hospital (BCH) in Beira (Mozambique). The study was approved by the Clinical Board of BCH (January 30, 2018), which waived the need for written informed consent given the retrospective nature of the study and the use of anonymized data from hospital records. Research was performed in accordance with relevant guidelines and regulations.

BCH is located in the province of Sofala, Mozambique and is the referral hospital for a geographical area that covers about 1.7 million people. In the province, average temperature ranges from 21 °C in July to 28.5 °C in January; the hot (and rainy) season runs approximately from November to March, and the cold season from June to August. About 5000 deliveries and 2100 admissions to the SCU occur every year at BCH. Medical transport system was not available in the province and outborn infants were brought to the hospital by their families.

### Patients

All neonates admitted to the SCU between January 1 and December 31, 2017 were evaluated for inclusion in the study. All neonates were included in (1) the assessment of availability of temperature at admission, and (2) the comparison of patient characteristics according to availability of temperature at admission. Then, only neonates with available temperature at admission were included in the analysis of mortality.

### Data collection

All data were retrieved from hospital records by hospital staff and were collected in an anonymized dataset. Maternal data included maternal age, HIV infection, twin pregnancy and number of previous gestations. Neonatal data included mode of delivery, birthplace, gestational age, sex, birth weight, 5-min Apgar score (for inborns), time to transfer and admission to SCU, diagnosis at admission, temperature at admission, and mortality through facility discharge.

Diagnosis at admission was based on clinical examination because availability of laboratory and instrumental exams was limited^18^.

At admission to SCU, neonatal axillary temperature was measured by the attending nurse using a digital thermometer (C202; Terumo, Tokyo, Japan). The nurse repeated the measurement of temperature in case of extreme hypothermia (< 35 °C) or extreme hyperthermia (> 39 °C). Severe/moderate hypothermia was defined as temperature < 36 °C, mild hypothermia as 36–36.4 °C, normal temperature as 36.5–37.5 °C and hyperthermia as > 37.5 °C^13^.

### Statistical analysis

Continuous data were reported as median and interquartile range (IQR), while categorical data as number and percentage.

The comparison of included (available temperature at admission) and excluded (unavailable temperature at admission) neonates was performed using Mann–Whitney test (continuous data) and Chi-square test (categorical data).

The relationship between mortality rate and neonatal temperature at admission was investigated with logistic regression models where temperature was modeled with first order polynomial or restricted cubic splines. The number of knots for the temperature was chosen in order to maximize the model likelihood ratio.

In all neonates (inborn, outborn, homebirth), a logistic regression model was estimated to assess the effect of admission temperature (modeled with restricted cubic splines) on mortality, adjusting for a set of pre-defined clinically relevant factors (diagnosis, place and mode of delivery, seasonality, sex, birthweight, twin birth, maternal age, HIV, and number of previous gestations). Model selection was performed by AIC reduction.

In the subgroup of inborn/outborn neonates, the 5-min Apgar score was added among the factors of the logistic regression model. In fact, the 5-min Apgar score is known to be a clinically relevant predictor of mortality, but it is not available in neonates born at home. Model selection was performed by AIC reduction.

Model performance was evaluated with internal validation (c-index) and calibration (calibration-in-the-large and calibration slope) using bootstrap methods (re-sampling with replacement to create 1000 samples of the same size as the original)^19^.

The results from the two regression models were summarized in two nomograms (one for all admitted neonates and one for inborn/outborn neonates).

All tests were 2-sided and a *p* value less than 0.05 was considered statistically significant. Statistical analysis was performed using R 3.5 (R Foundation for Statistical Computing, Vienna, Austria)^20^.

## Results

From 1st January to 31st December 2017, 2098 neonates were admitted to SCU for prematurity (648, 30.9%), asphyxia/HIE (559, 26.6%), wet lung (216, 10.3%), trauma (15, 0.7%), congenital malformations (96, 4.6%), fever (67, 3.2%), sepsis (55, 2.6%), seizures (17, 0.8%), jaundice/hyperbilirubinemia (34, 1.6%), or with other diagnoses (381, 18.2%). Neonatal temperature at admission was available in 1344 neonates (64%), who were included in the analysis. The comparison of 1344 included neonates (available temperature at admission) and 754 excluded neonates (unavailable temperature at admission) neonates is reported in Table 1. Birth weight (*p* = 0.004), 5-min Apgar score (*p* = 0.006), birthplace (*p* = 0.02) and diagnosis at admission (*p* = 0.003) were different between included and excluded neonates (Table 1). Mortality rate was 36.5% in included neonates and 30.5% in excluded neonates (*p* = 0.007). Among inborns, resuscitation in delivery room was needed in 334 out of 746 included subjects (44.8%) and in 91 out of 193 excluded subjects (57.2%; information not available in 267 outborns) (*p* = 0.61). Time from birth to admission was not different between included and excluded neonates (*p* = 0.82, Table 1). Maternal age, number of gestation and HIV infection were not different between included and excluded neonates (Table 1).

**Table 1:** Comparison of included (available temperature at admission) and excluded (unavailable temperature at admission) neonates.

	Included neonates (available temperature at admission)	Excluded neonates (unavailable temperature at admission)	*p* value
No. of subjects	1344	754	–
**Neonates**			
Gestational age (weeks)^a,b^	37 (34–39)	37 (33–39)	0.20
Sex male:female^c^	752:591	430:313	0.43
Birth weight (g)^a,d^	2450 (1700–3050)	2600 (1850–3100)	0.004
Twin^e^	263 (19.6)	73 (19.9)	0.94
5-min Apgar score^a,f^	8 (6–9)	8 (6–8)	0.006
**Mode of delivery**^g^			0.91
Vaginal	995 (75.4)	264 (75.0)	
Caesarean	340 (24.6)	88 (25.0)	
**Birthplace**^h^			0.02
Inborn	746 (55.5)	460 (61.8)	
Outborn	499 (37.1)	234 (31.5)	
Homebirth	99 (7.4)	50 (6.7)	
Total time from birth to admission (min)^a,i^	61 (39–194)	66 (40–134)	0.82
**Diagnosis at admission**			0.003
Asphyxia/HIE	356 (26.5)	203 (26.9)	
Prematurity/LBW	441 (32.8)	207 (27.5)	
Sepsis	42 (3.1)	13 (1.7)	
Fever	53 (3.9)	15 (2.0)	
Congenital malformations	54 (4.0)	42 (5.6)	
Wet lung	123 (9.1)	93 (12.3)	
Trauma	10 (0.7)	5 (0.7)	
Seizures	12 (0.9)	5 (0.7)	
Jaundice/ hyperbilirubinemia	25 (1.9)	9 (1.2)	
Other diagnoses	229 (17.0)	162 (21.5)	
**Mothers**			
Maternal age (years)^a,j^	23 (19–28)	23 (20–28)	0.98
Number of previous gestations (n)^a,k^	2 (1–4)	2 (1–3)	0.55
HIV positive mothers^l^	327 (25.5)	98 (20.2)	0.19
Mortality^m^	488 (36.5)	215 (30.5)	0.007

*HIE* Hypoxic ischemic encephalopathy, *LBW* low birth weight.

Data expressed as No. (%) or ^a^median (IQR).

Data not available in ^b^504, ^c^12, ^d^14, ^e^387, ^f^233, ^g^411, ^h^10, ^i^1021, ^j^472, ^k^438, ^l^478, and ^m^56 subjects.

**Figure 1:**
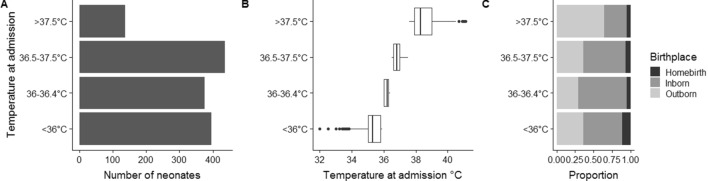
Number of neonates (**A**), boxplot of neonatal temperature (**B**) and birthplace (**C**) according to ranges of neonatal temperature at admission.

Among 1344 included neonates, median temperature at admission was 36.3 °C (IQR 35.8–36.9 °C; min 32 °C, max 41.1 °C). Severe/moderate hypothermia was reported in 395 neonates (29.4%), mild hypothermia in 376 (28.0%), normal temperature in 436 (32.4%) and hyperthermia in 137 (10.2%) (Fig. 1A,B). Birthplace was associated with neonatal temperature at admission (*p* < 0.0001): the highest proportion of homebirths was found in severely/moderately hypothermic neonates, while the highest proportion of outborn was found in neonates with hyperthermia (Fig. 1C).

**Figure 2:**
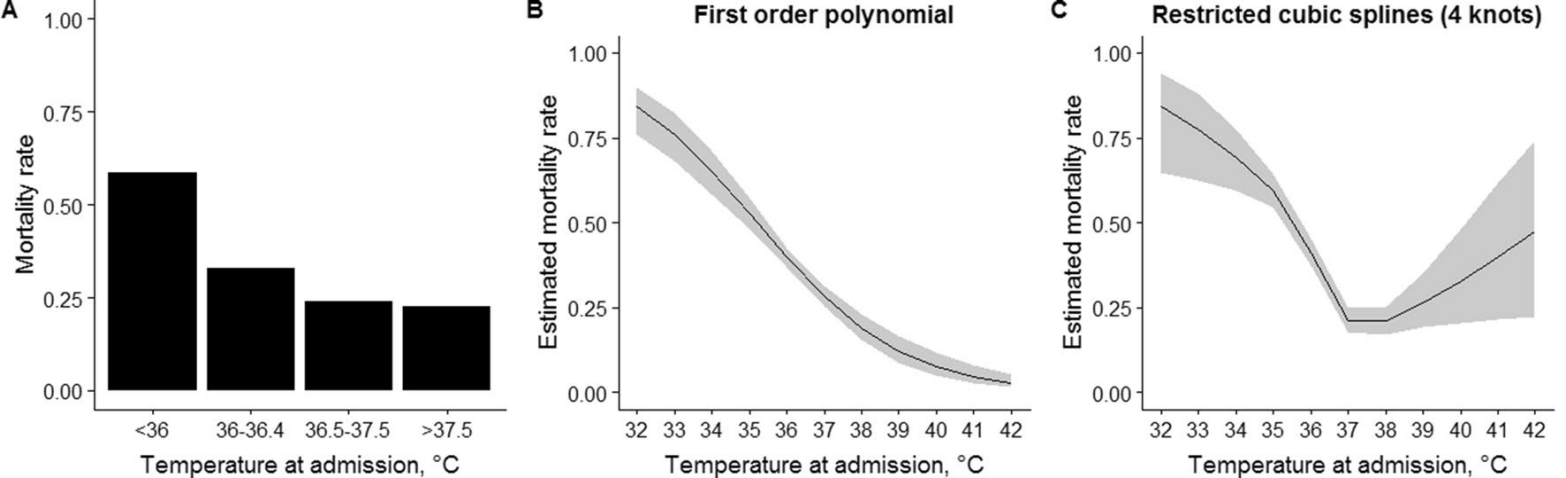
Observed mortality rate according to ranges of neonatal temperature at admission (**A**); estimated mortality rate according to neonatal temperature at admission as modeled with first order polynomial (**B**) or restricted cubic splines (**C**). Shaded areas represent bootstrap 95% confidence intervals.

**Figure 3:**
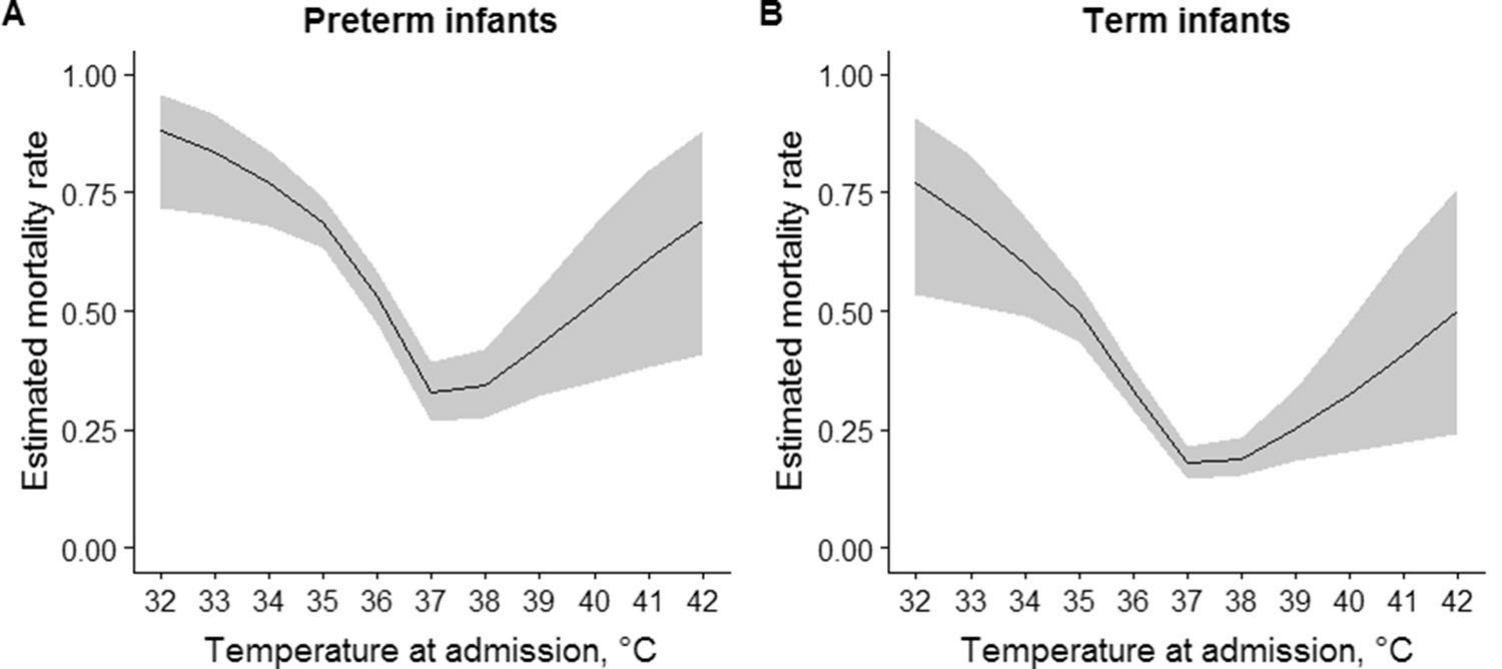
Non-linear association between mortality rate and temperature in preterm (**A**) and term (**B**) infants. Shaded areas represent bootstrap 95% confidence intervals.

After a median length of stay of 4 days (IQR 2–8), 848 neonates were discharged alive and 488 died, while the information was not available in eight neonates. Observed mortality rate was 58% (231 out of 395) in severe/moderate hypothermia, 33% (123 out of 372) in mild hypothermia, 24% (103 out of 432) in normal temperature range and 23% (31 out of 137) in hyperthermia (Fig. 2A). The relationship between mortality rate and neonatal temperature at admission was investigated with logistic regression models where temperature was modeled with first order polynomial and with restricted cubic splines (Fig. 2B,C). A non-linear association between mortality rate and temperature was identified (non-linear term: *p* < 0.0001), thus the model with cubic splines was preferred over the model with first order polynomial. Four knots were identified in 34.6 °C, 36 °C, 36.6 °C and 38.3 °C. Estimated mortality rate decreased from 84% at 32 °C to 64% at 34.6 °C (mean − 8% per °C), to 41% at 36 °C (mean − 16% per °C), to 26% to 36.6 °C (mean − 25% per °C) and to 22% at 38.3 °C (mean − 2% per °C), then increased to 40% at 41 °C (mean + 7% per °C). Mortality rate was estimated to be at a minimum at admission temperature of 37.5 °C (Fig. 2C). The curve of estimated mortality rate according to admission temperature had similar shape in term and preterm infants (*p* = 0.78), but the latter had higher mortality rate (*p* < 0.0001) (Fig. 3).

Since information on gestational age was largely missing, multivariable analysis included the diagnosis of prematurity as proxy of the role of gestational age, although prematurity underlines a wide range of gestational ages. Since all neonates born from caesarean sections were inborns, birthplace and mode of delivery were collapsed in one variable with four categories (caesarean inborn, vaginal inborn, vaginal outborn and vaginal homebirth).

**Figure 4:**
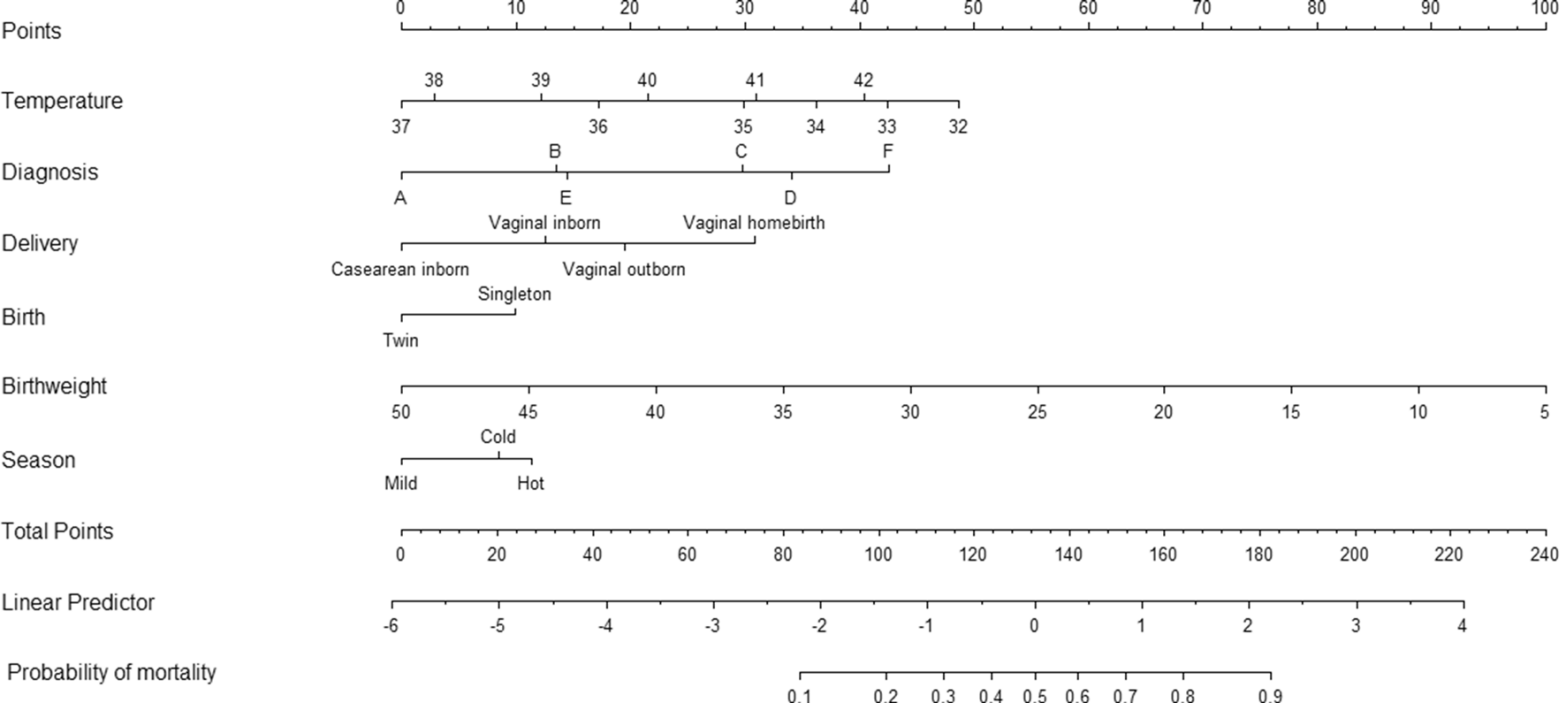
Nomograms for all patients. Diagnosis legend: fever, trauma, jaundice/hyperbilirubinemia, other (**A**); wet lung (**B**); asphyxia/HIE (**C**); sepsis/seizures (**D**); prematurity (**E**); congenital malformations (**F**). Neonatal temperature at admission is expressed in °C. Birthweight is expressed as 100 g.

In all infants (inborns, outborns, homebirths), admission temperature (linear term *p* < 0.0001, non-linear term *p* < 0.0001), diagnosis (*p* < 0.0001), delivery (*p* < 0.0001), birthweight (*p* < 0.0001), twin birth (*p* = 0.01) and seasonality (*p* < 0.0001) were included in the final model. Internal validation and calibration via bootstrapping showed good validation (c-index 0.78) and calibration (calibration-in-the-large − 0.0264 and calibration slope 0.9530). The model was graphically represented by the nomogram in Fig. 4.

**Figure 5:**
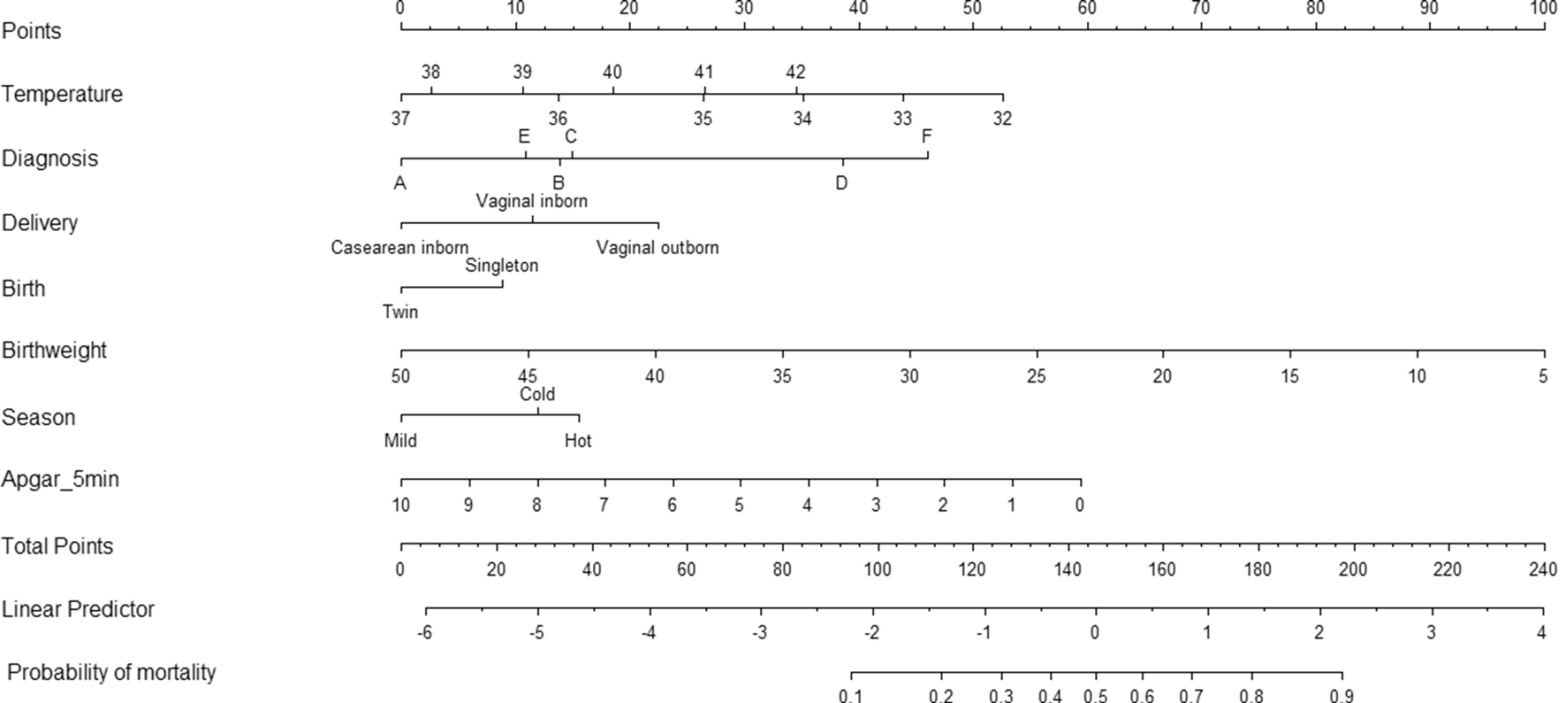
Nomograms for inborn and outborn patients. Diagnosis legend: fever, trauma, jaundice/hyperbilirubinemia, other (**A**); wet lung (**B**); asphyxia/HIE (**C**); sepsis/seizures (**D**); prematurity (**E**); congenital malformations (**F**). Neonatal temperature at admission is expressed in °C. Birthweight is expressed as 100 g.

In inborn/outborn infants, admission temperature (linear term *p* < 0.0001, non-linear term *p* = 0.0002), diagnosis (*p* < 0.0001), delivery (*p* < 0.0001), 5-min Apgar score (*p* < 0.0001) birthweight (*p* < 0.0001), twin birth (*p* = 0.01) and seasonality (*p* < 0.0001) were included in the final model. Internal validation and calibration via bootstrapping showed good validation (c-index 0.79) and calibration (calibration-in-the-large − 0.0286 and calibration slope 0.9407). The model was graphically represented by the nomogram in Fig. 5.

Information on both models is reported in Supplementary Table 1.

## Discussion

Our findings highlighted a substantial underreporting of neonatal temperature at admission, but also showed that the majority of neonates were admitted with hypothermia. Neonatal temperature at admission had a non-linear relationship with mortality, which was the lowest rate at 37.5 °C and increased with different slopes when departing from the interval 36.6–38.3 °C.

To our knowledge, this is the first study to investigate the differences in mortality risk across a full range of admission temperatures and to describe a non-linear relationship between neonatal temperature at admission and mortality in a low-resource setting. Our findings confirm that both hypothermia and hyperthermia at admission are associated with mortality, but add a differentiation of mortality risk according to the specific temperature.

The study has some limitations. First, it is a retrospective study, thus quality of data was limited. For example, information on gestational age was largely missing (a common problem in low-resource settings), thus the model included the diagnosis of prematurity as proxy of the role of gestational age, although prematurity underlines a wide range of gestational weeks. Second, temperature at admission was not available in about one third of neonates. Third, diagnosis at admission was based on clinical examination because availability of laboratory and instrumental exams was limited.

Both hypothermia and hyperthermia represent important predictors of neonatal morbidity and mortality^1,2^. However, neonatal temperature is often missing in clinical records although being an important and easy-to-measure indicator^3^. Our findings showed that admission temperature was not available in around one out of three neonates, thus highlighting the substantial underreporting of such indicator. Since neonatal temperature at admission can be used as quick feedback of neonatal care during the immediate postnatal phase, the lack of such data hampers the implementation of quality improvement processes^2^.

In low resource settings, the incidence of postnatal hypothermia is very high, ranging from 32 to 85%^3^, while hyperthermia at admission has received less attention^1,3,6^. Our data indicated that around 57% of neonates were admitted with hypothermia, thus confirming that prevention of postnatal thermal losses is still an underappreciated major challenge in low resource settings^3^.

Available literature approached the investigation on neonatal temperature based on the few categories indicated by the WHO classification^3,6,13^. This approach offers the advantages of easy interpretation and application in clinical practice, but constrains the variability of the phenomenon into pre-specified classes^14^. A recent study attempted to overcome such disadvantage by splitting admission temperature in a larger number of categories and suggested a U-shaped relationship between admission temperature and adverse neonatal outcomes^15^. The U-shape relationship implicated that both low and high temperatures impaired neonatal outcomes, but the generalizability of these findings was limited by the inclusion of very preterm infants, the high-resource setting and the categorization of the temperature^15^. Our study adds the information on the differences in mortality risk across a full range of admission temperatures, in both preterm and term infants born in a low-resource setting. In addition, our findings displays the relationship between mortality and temperature as asymmetric inverted omega-shaped curve rather than U-shaped curve. Such non-linear relationship confirmed that both low and high temperatures increased the mortality risk, but highlighted the different slopes when departing from normothermia (Fig. 2C).

These findings reinforce the need for temperature assessment in all admitted neonates and confirm the worse impact on mortality of low temperatures compared to high temperatures in a low-resource setting. Moreover, this approach allows the definition of a personalized risk assessment that goes beyond the categorical approach. Our results are summarized in two nomograms (one for all admitted neonates and one for inborn/outborn neonates) that can be considered among the proposals for the development of a quick mortality-risk calculator to be used in clinical practice. The nomograms require external validation in order to assess their generalizability to other low-resource settings. If confirmed, the nomograms can be transformed in on-line tools or apps for an easy and quick assessment of personalized mortality risk in clinical practice. Available literature offers other neonatal mortality risk scoring systems, such as CRIB-II, SNAP, NMS, NMR-2000^21–24^. Unfortunately, we could not use such scores because they include some parameters that were not available in the care (hence in the hospital records) of our study setting (i.e. blood gas analysis, oxygen saturation, mechanical ventilation). In addition, some of them were developed for specific categories of neonates (i.e. birthweight < 1500 g or gestational age < 31 weeks; birthweight < 2000 g).

Neonatal temperature at admission should be recorded as prognostic factor as well as quality indicator^2^. In the present study, the underreporting of such indicator highlights the need for enhancing the awareness of the importance of including temperature measurement among routine care. Appropriate actions may include audits (to identify local reasons for the underreporting), education to health care givers who are involved in neonatal care at admission (to enhance awareness of the importance of neonatal temperature), and continuous feedback on this quality improvement (to monitor the implementation).

Furthermore, the high rate of hypothermia and hyperthermia at admission calls for implementation of adequate strategies to ensure normothermia immediately after birth in low-resource settings^3,17^. Quality improvement activities should focus on enlisting thermal stability (i.e. warm delivery room, immediate drying, skin-to-skin contact, early breastfeeding, delayed bathing, adequate clothing, warm transport, keeping baby close to the mother) among the central objectives in management protocols in low-resource settings^6,13^. Provider training, enhancing awareness and revision of management routines seem to be the key elements for achieving such goal^3,6,13,17^.

## Conclusions

In a low-resource setting, neonatal temperature at admission showed a non-linear relationship with mortality rate, with different impacts of low and high temperatures. Only 32% of the infants were normothermic at admission, indicating the need for implementation of adequate strategies to enhance thermal stability. Temperature at admission was not reported in 36% of the infants, suggesting the need for more closely monitoring the admission temperature.

## Supplementary information

Supplementary Information.
