# Supplementary material for: Non-linear association between admission temperature and neonatal mortality in a low-resource setting
Source: Sci Rep. 2020 Nov 27;10:20800. doi: 10.1038/s41598-020-77778-5 (PMC7695844; doi:10.1038/s41598-020-77778-5)
Supplement: Supplementary file 1 — Supplementary Information. [file 41598_2020_77778_MOESM1_ESM.docx]

**TITLE: Non-linear association between admission temperature and neonatal mortality in a low-resource setting**

**Authors:** Francesco Cavallin, MSc ^a^, Serena Calgaro, MD ^b,c^, Valentina Brugnolaro, MD ^c^, Olivier Manzungu Wingi, MD ^d^, Arlindo Rosario Muhelo, MD ^d^, Liviana Da Dalt, MD ^c^, Damiano Pizzol, MD ^b^, Giovanni Putoto, MD ^b^, Daniele Trevisanuto, MD ^c^

**Institutions:**

^a^ Independent statistician, Solagna, Italy; ^b^ Doctors with Africa CUAMM, Padova, Italy;

^c^ Department of Woman’s and Child’s Health, University of Padova, Padova, Italy; ^d^ Central Hospital of Beira, Beira, Mozambique

**Address correspondence to:** Professor Daniele Trevisanuto, MD, Department of Women and Children Health, University of Padova, Via Giustiniani, 3, 35128, Padova, Italy,

e-mail: [daniele.trevisanuto@unipd.it](mailto:daniele.trevisanuto@unipd.it).

**Funding Source.** This study was funded by a contribution of the of Italian Agency for Development Cooperation (grant AID 11497/CUAMM/MOZ - Healthy Newborn Project: Innovative approaches in protecting the health of the newborn in the Province of Sofala).

**Financial disclosure.** The authors have no financial relationship relevant to this article to disclose.

**Conflict of Interest.** The authors have no potential conflicts of interest to disclose.

**SUPPLEMENTARY MATERIAL**

Supplementary Table 1. Multivariable analysis of mortality

|  |  | All infants | | | Inborn and outborn infants | | |
| --- | --- | --- | --- | --- | --- | --- | --- |
|  |  | Chi-square | Degrees of freedom | p-value | Chi-square | Degrees of freedom | p-value |
| Variables in the model | Temperature at admission  Linear term  Non-linear term | 50.60  26.48 | 3  2 | <0.0001  <0.0001 | 31.76  16.38 | 3  2 | <0.0001  0.0003 |
|  | Diagnosis | 52.20 | 5 | <0.0001 | 33.47 | 5 | <0.0001 |
|  | Delivery | 31.61 | 3 | <0.0001 | 24.78 | 2 | <0.0001 |
|  | Birthweight | 52.23 | 1 | <0.0001 | 46.37 | 1 | <0.0001 |
|  | Twin birth | 6.51 | 1 | 0.01 | 3.78 | 1 | 0.05 |
|  | Seasonality | 10.90 | 2 | 0.004 | 15.72 | 2 | 0.0004 |
|  | 5-minute Apgar score | - | - | - | 33.95 | 1 | <0.0001 |
| Variables not in the model | HIV | 0.54 | 1 | 0.46 | 0.06 | 1 | 0.81 |
|  | Sex | 2.22 | 1 | 0.14 | 2.72 | 1 | 0.10 |
|  | Maternal age | 0.55 | 1 | 0.46 | 1.29 | 1 | 0.26 |
|  | Number of previous gestations | 0.94 | 1 | 0.33 | 0.39 | 1 | 0.81 |
